# Calretinin and parvalbumin in schizophrenia and affective disorders: a mini-review, a perspective on the evolutionary role of calretinin in schizophrenia, and a preliminary post-mortem study of calretinin in the septal nuclei

**DOI:** 10.3389/fncel.2015.00393

**Published:** 2015-10-29

**Authors:** Ralf Brisch, Hendrik Bielau, Arthur Saniotis, Rainer Wolf, Bernhard Bogerts, Dieter Krell, Johann Steiner, Katharina Braun, Marta Krzyżanowska, Maciej Krzyżanowski, Zbigniew Jankowski, Michał Kaliszan, Hans-Gert Bernstein, Tomasz Gos

**Affiliations:** ^1^Department of Forensic Medicine, Medical University of GdańskGdańsk, Poland; ^2^Department of Psychiatry and Psychotherapy, Otto-von-Guericke-University of MagdeburgMagdeburg, Germany; ^3^School of Medicine, The University of AdelaideAdelaide, SA, Australia; ^4^Institute of Evolutionary Medicine, University of ZurichZurich, Switzerland; ^5^Department of Psychiatry and Psychotherapy, Ruhr University BochumBochum, Germany; ^6^Center for Behavioral Brain SciencesMagdeburg, Germany; ^7^Department of Zoology/Developmental Neurobiology, Institute of Biology, Otto-von-Guericke-University of MagdeburgMagdeburg, Germany

**Keywords:** calretinin, parvalbumin, septal nuclei, post-mortem studies, schizophrenia, affective disorders, evolution of the human brain

## Abstract

**Objective:** The septal nuclei are important limbic regions that are involved in emotional behavior and connect to various brain regions such as the habenular complex. Both the septal nuclei and the habenular complex are involved in the pathology of schizophrenia and affective disorders.

**Methods:** We characterized the number and density of calretinin-immunoreactive neurons in the lateral, medial, and dorsal subregions of the septal nuclei in three groups of subjects: healthy control subjects (*N* = 6), patients with schizophrenia (*N* = 10), and patients with affective disorders (*N* = 6).

**Results:** Our mini-review of the combined role of calretinin and parvalbumin in schizophrenia and affective disorders summarizes 23 studies. We did not observe significant differences in the numbers of calretinin-immunoreactive neurons or neuronal densities in the lateral, medial, and dorsal septal nuclei of patients with schizophrenia or patients with affective disorders compared to healthy control subjects.

**Conclusions:** Most post-mortem investigations of patients with schizophrenia have indicated significant abnormalities of parvalbumin-immunoreactive neurons in various brain regions including the hippocampus, the anterior cingulate cortex, and the prefrontal cortex in schizophrenia. This study also provides an explanation from an evolutionary perspective for why calretinin is affected in schizophrenia.

## Introduction

Ca^2+^-binding proteins (CBPs) are classified as Ca^2+^-puffer proteins (CPPs; parvalbumin, calretinin, calbindin, calcineurin, and the S100 family) or Ca^2+^-sensor proteins (CSSs; calmodulin, and VILIP-1,3). The processes by which CPPs and CSSs interact are not well-understood. In schizophrenia, CBPs are used to identify altered GABAergic (γ-aminobutyric acid-producing) interneurons in various brain regions, including the prefrontal cortex, hippocampus, and amygdala (Inan et al., 2013). Calretinin belongs to a subset of inhibitory interneurons that uses the neurotransmitter GABA (Barinka and Druga, 2010; Cauli et al., 2014). GABA is converted by the action of glutamic acid decarboxylase (GAD), which exists as two isoforms, GAD65 and GAD67. GAD65 is localized in axon terminals, and GAD67 is localized in neuronal cell bodies (Blum and Mann, 2002). Calretinin has been detected in the cingulate and entorhinal cortices as well as the hippocampus, brainstem, and cerebellum in the human brain (Nitsch and Ohm, 1995; Mikkonen et al., 1997; Baizer, 2014). White matter interneurons express GABA, calbindin, and calretinin (Suárez-Solá et al., 2009), which are relevant in schizophrenia (review by Kostovic et al., 2011; Yang et al., 2011; Joshi et al., 2012).

In the present study, we assessed the presence of calretinin in the anterior, middle, and posterior portions of the human septal nuclei. Recent findings regarding the involvement of calretinin in neurogenesis and animal models of psychiatric diseases imply that it is important to study the role of calretinin in the septal nuclei in schizophrenia and affective disorders. A loss of calretinin causes a deficit in adult hippocampal neurogenesis (Todkar et al., 2012). In adult mice, calretinin-positive neurons are present in the dentate gyrus, an important neurogenic zone (Spampanato et al., 2012). The transcription factor Gsx2 (genetic screened homebox 2) proliferates in the human cortical subventricular zone and commits cortical stem cells into calretinin-expressing cells (Radonjic et al., 2014). Calretinin cells originate from the subventricular zone of the lateral and caudal ganglion eminences (González-Gómez and Meyer, 2014). The density of calretinin-positive neuronal progenitors along the septo-temporal axis of the hippocampus was decreased by unpredictable chronic mild stress (UCMS), and this effect was inhibited by the treatment with the antidepressant fluoxetine (Tanti et al., 2013). Alterations in calretinin expression have been observed in mouse models of epilepsy and psychiatric diseases (Shin et al., 2013); however, no significant differences in the number of calretinin-immunoreactive interneurons in the cerebral cortices of wild-type and DBZ (DISC1-binding zinc finger protein) knockout (KO) mice have been reported (Koyama et al., 2013). Electroconvulsive therapy (ECT) results in the neurogenesis of calretinin-positive interneurons (Inta et al., 2013). The septum, via cholinergic and GABAergic pathways, is involved in the regulation of mesolimbic dopamine transmission (Lecourtier et al., 2010). The suppression of the septohippocampal pathway and its GABAergic activity might represent a novel treatment for the symptoms of schizophrenia (Ma et al., 2012; Deidda et al., 2014). The medial habenular complex is connected to the septal nuclei through the stria medullaris (Sutherland, 1982; Hikosaka, 2013). Habenular dysfunction is involved in schizophrenia (Heldt and Ressler, 2006), and habenular calcification has been reported in schizophrenia (Sandyk, 1992). A reduction in the volume of the medial and lateral habenular complex and reductions in the cell number and area of the medial habenula have been observed in affective disorders (Ranft et al., 2010), mainly a habenular volume reduction in unmedicated bipolar patients (Savitz et al., 2011a), but not in patients with post-traumatic stress disorder (Savitz et al., 2011b). Further, diminished neuronal density has been reported in the lateral septal nucleus of brain sections from bipolar patients compared with control subjects using both Nissl and Heidenhain-Woelke methods (Brisch et al., 2011).

The aim of this sudy is to investigate whether alterations of calretinin-immunoreactive neurons exist in the lateral, medial and dorsal septal nuclei in patients with schizophrenia and patients with affective disorders in comparison with healthy control subjects, based on the pathway between the septal nuclei and the habenular complex and the importance of calretinin in neurogenesis and animal models of psychiatric diseases.

## Experimental procedures

### Subjects

All brains used in this study were from the Brain Collection of the University of Magdeburg. Brains were obtained from pathologists or medical examiner offices in the years 1987–2002 according to the Declaration of Helsinki (1975) and German and EU laws and after approval by the university's ethic commission. The mean demographic data for all individual cases (all were Caucasian) including brain weight, post-mortem delay, onset of disease, and duration of disease are present in Table 1. The three groups were carefully matched for gender, age, post-mortem delay, the age at onset of illness, and brain weight. The post-mortem brains of six subjects lacking any signs of neurological or psychiatric symptoms were used as a control group. Brains from 10 patients with schizophrenia diagnosed according to the DSM-IV (Diagnostic and statistical manual of mental disorders) and ICD-10 (International statistical classification of diseases and related health problems) criteria were included; most of these patients had received antipsychotic treatment for at least several years. In addition, the brains of six patients with affective disorders according to DSM-IV and ICD-10 were studied. Of these, three patients were diagnosed with bipolar disorder (DSM IV-TR: 296.5; F 31.3) with manic and depressive episodes, and three patients suffered from major depressive disorder (DSM IV-TR: 296.5; F 31.5). All patients with affective disorders had received mood stabilizers consistently or periodically and/or antidepressive medication for several years before death. Only patients with detailed clinical records and well-documented psychopathology were included. The criteria for exclusion from the three groups were as follows: (i) organic brain disease; (ii) brain injury; (iii) alcoholism or chronic substance abuse; (iv) chronic somatic diseases affecting the central nervous system (i.e., cachexia, cancer, chronic liver or kidney diseases, or long term corticosteroid treatment); and (v) age greater than 65 years, to exclude changes related to normal aging of the brain.

**Table 1:** **Demographic data and group parameters for healthy control subjects, patients with affective disorders, and patients with schizophrenia**.

	**Control subjects**	**Affective disorders**	**Schizophrenia**
*N*	6	6	10
Males/Females	2∕4	2∕4	6∕4
Age (years)	52.7±9.7	48.7±11.6	54.8±8.9
Brain weight (g)	1298.3±169.6	1373.3±155.9	1305.7±155.2
Brain volume (cm^3^)	1252.0±163.6	1324.3±150.3	1259.1±149.7
Post-mortem delay (h)	36.0±20.1	29.2±14.7	31.7±15.2
Duration of illness (years)		9.7±6.8	23.7±12.9
Onset of illness (years)		39.0±10.1	31.1±11.1
Thickness of section (μm)	16.8±1.2	16.9±1.8	14.9±1.9

*Mean ± standard deviation*.

### Tissue processing

Brains were removed within 4–72 h after death (see Table 1 for the demographic data of control subjects and patients) and fixed in toto in 8% phosphate-buffered formaldehyde for at least 2 months (pH = 7.0, *T* = 15–20°C). The frontal and occipital poles were separated by coronal cuts anterior to the genu and posterior to the splenium of the corpus callosum. After embedding all parts of the brain in paraffin, serial whole brain sections without midline cut of the middle block were cut (20 μm) with a calibrated microtome and mounted. The shrinkage factor caused by fixation and embedding and the thickness of the slices were calculated by methods described previously by Baumann et al. (1999). The mean volume shrinkage factor for brains in the schizophrenia, affective disorder, and control groups was 2.2 ± 0.3 (mean ± SD). No significant differences in the shrinkage factors were observed among the three groups. Every 50th section was stained for calretinin. The distance between the sections was 1 mm.

### Stereological-based analysis and morphometric delineation criteria

**Figure 1:**
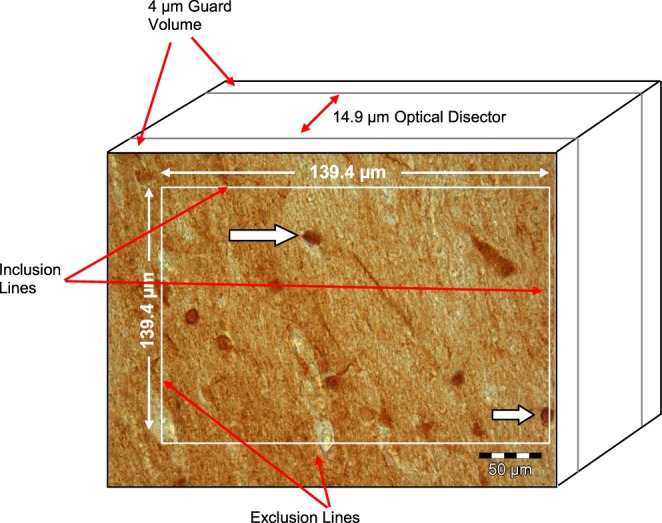
**Calretinin-immunopostive neurons (arrows) in the Ncl. lateralis of a patient with schizophrenia (modified from Pennington et al., 2008)**.

For the present study, one coronal sections was randomly selected from each brain. Each section was located at the same clearly defined anatomical landmarks in either the anterior, middle, or posterior portion of the human septum. The cross-sectionals areas of the septal nuclei within each section were determined using a computerized image system (Digitrace Imaging System). The borders of the septal tissue were delineated under a microscope at low magnification with a 2.5 × objective according to the boundaries described by Horváth and Palkovits (1987). The anterior border of the septal tissue is the genu of the corpus callosum; the upper border is the body of the corpus callosum and the anterior commissure; and the lateral borders are the lateral ventricles. The septal tissue is surrounded basally by the nucleus accumbens and the stria terminalis. To determine interrater reliability, stereological measurements of eight different, randomly selected brains were performed by two investigators (R.B., R.S). The interrater reliability for the densities of calretinin-immuno-positive neurons in the septal nuclei was 0.97 (intraclass correlation coefficient). All measurements were performed blind to the diagnosis: the investigators were unaware of the patient's diagnosis, age, and gender. The cross-sectional area of the section was scanned with a 2.5 × objective using a video camera module attached to a Leica light microscope, and Digitrace software was used to project a picture on a monitor (22.0 × 15.9 mm). A magnification of 400 × was used for cell counting. Using this apparatus, the counting frame was superimposed onto one section at clearly defined anatomical landmarks, with up to 200 systemically, uniformly randomly sampled counting boxes (i.e., up to 100 counting boxes for the left and the right portions of the septal nuclei) for each septal nucleus along the entire extent of the septal nucleus. The actual section thickness of each section in the septal nuclei was determined with a 100 × oil immersion objective by focusing on the upper and lower surfaces of the section and then subtracting the z-axis distance measured by the a microcator attached to the Leica DM RB microscope (Leica, Gieen, Germany). To determine the number of neurons at a higher magnification (400X) neurons were counted by using the optical disector method as described earlier (Bernstein et al., 2001; Brisch et al., 2009; Walløe et al., 2014). The average thickness of the sections (z-axis) was 16.0 ± 1.9 μm (mean ± SD). The mean thickness of the sections was 14.9 ± 1.9 μm (mean ± SD) in the schizophrenia group, 16.9 ± 1.8 μm (mean ± SD) in the affective disorders group, and 16.8 ± 1.2 μm (mean ± SD) among healthy control subjects. The neuronal density was estimated based on the square of the counting area, which was determined by the square of the septal nuclei at the adjacent nuclei, and the number of calretinin-immunoreactive neurons within the counting boxes (Brisch et al., 2009). Neurons touching the left and lower borders of the counting boxes were excluded, and neurons touching the opposite borders were included (see Figure 1; Pennington et al., 2008).

### Immunohistochemistry

The brain sections were dewaxed with xylol and washed with distilled water (two 10-min washes). The sections were washed in a 1% H_2_O_2_–10% methanol/phosphate-buffered saline solution. After repeated washings in phosphate-buffered saline solution, the sections were incubated in bovine serum albumin in a humidified chamber for 1 h. A rabbit polyclonal antiserum (Swant, Bellizona, Switzerland, Code No: 7699/4) was then applied to the brain sections as the primary antibody in a dilution of 1:500. The solution consisted of 20 μl of calretinin antibody, 10 ml of PBS, 40 μl of Triton X-100, and 200 ml of goat normal serum. The brain sections were incubated in a humidified chamber at 4°C for 48 h and washed twice for 10 min each in phosphate-buffered saline. The secondary antibody (goat-anti-rabbit-immunoglobulin E 0432, Dako, Denmark) was then applied to the sections at a dilution of 1:100, and the sections were incubated in a humidified chamber for 48 h, followed by two washes for 5 min each in phosphate-buffered saline. Streptavidin was then applied to the sections at a dilution of 1:100 (Streptavidin-biotin-peroxidase-complex, RPN 1051, Batch 177351, Amersham Biosciences, Germany). Streptavidin was used as an antibody marker. The sections were then incubated in a humidified chamber at a 4°C for 1 h, followed by two washes for 10 min each in phosphate-buffered saline. To visualize the reaction products, 3,3′-diamino nickel sulfate hexahydrate was used. Finally, repeated washings with distilled water (twice for 5 min), 60% alcohol (5 min), 70% alcohol (5 min), 96% alcohol (5 min), and absolute alcohol (5 min) were performed. To control the specificity of the immunostaining for calretinin, we either omitted the primary antiserum or replaced it with buffer or normal rabbit serum. Control reactions showed a complete disappearance of specific immunostaining.

### Statistical analysis

The independence of frequency for the variables of gender and diagnosis was analyzed using Pearson's chi-square test. The other demographic variables are presented as their mean ± standard deviation and were compared among the three groups using a one-way ANOVA (analysis of variance; see Table 1). Levene's-test was used to evaluate the equality of variances for a given variable, such as the cell numbers calculated for the three groups (i.e., healthy control subjects, patients with affective disorders, and patients with schizophrenia). Some of the morphometric values had distinct asymmetric distributions (see Table 2) and were therefore using non-parametric tests. The Kruskal–Wallis test was performed to determine the significance of the differences in terms of mean cross-sectional area, the number of neurons, and the neuronal densities among the three groups (see Table 2). Results were considered significant at the 0.05 level. In cases of significance, the pairwise U-test (Wilcoxon–Mann–Whitney with the Shaffer-correction) was used to detect significant differences between pairwise groups (see Table 2). The statistical power of each test was also calculated.

**Table 2:** **Cell numbers and neuronal densities (neurons/mm^3^) in the septal nuclei of patients with schizophrenia, patients with affective disorders, and the healthy control subjects**.

**Cell number (mean, SD, CE)**	**Ncl. lateralis**	**Ncl. medialis (pars fimbrialis and pars intermedia)**	**Ncl. dorsalis**
**Volume cell density (mean, SD, CE)**			
Control subjects (*N* = 6)	123.0 (81.4; 0.27)	171.6 (85.7; 0.22) (*N* = 5)	131.0 (87.8; 0.27)
	2032 (687; 0.14)	1717 (684; 0.18)	1711 (1373; 0.33)
Patients with schizophrenia (*N* = 10)	257.4 (243.3; 0.32) (*N* = 9)	329.7 (374.1; 0.43) (*N* = 7)	224.2 (299.6; 0.45) (*N* = 9)
	2891 (1939; 0.22)	2863 (3452; 0.46)	2110 (2389; 0.38)
Patients with affective disorders (*N* = 6)	168.2 (91.3; 0.22)	165.4 (98.3; 0.27) (*N* = 5)	238.0 (150.5; 0.26)
	2378 (1193; 0.20)	1744 (730; 0.19)	2856 (1281; 0.18)
Two-group-comparisons			
Aff. vs. Ctr. (U-test)	0.48; 0.59	1.00; 1.00	0.24; 0.24
SZ vs. Ctr. (U-test)	0.18; 0.61	0.76; 0.88	0.69; 0.86
SZ vs. Aff. (U-test)	0.78; 0.78	0.88; 1.00	0.78; 0.22
Three-group-comparisons			
ANOVA Ctr./Aff./SZ (*p*-value)	0.34; 0.54	0.46; 0.62	0.65; 0.57
Levene's-test	**0.048**; **0.071**	**0.036**; 0.10	0.50; 0.44
K–W-test Ctr./Aff./SZ	0.38; 0.76	0.94; 0.97	0.56; 0.33

*The data are presented as the mean, standard deviation (SD), and coefficient of error (CE). Please note that the number of patients with schizophrenia included in the data for the nuclei lateralis, medialis (pars fimbrialis and pars intermedia), and dorsalis data is less than the original number of patients with schizophrenia (in bold). In addition, the number of patients with affective disorders included in the nuclei medialis data is less than the original number of patients with affective disorders (in bold). K–W-test, Kruskal–Wallis-test; Ctr, Control subjects; SZ, Patients with schizophrenia; Aff, Patients with affective disorders; Ncl., nucleus. Shrinkage-corrected data are presented*.

#### Methods of the mini-review

We searched PubMed in July 2015. Using the following keywords “calretinin and neuronal density and schizophrenia,” we obtained 12 hits, of which three were relevant studies. We had 18 hits in PubMed for the keywords “calretinin and neuronal number and schizophrenia,” two of which were relevant studies. We had two hits in PubMed for the keywords “calretinin and neuronal density and bipolar disorder,” one of which was a relevant study. We had two hits in PubMed for the keywords “calretinin and neuronal number and bipolar disorder,” one of which was a relevant study. We had two hits in PubMed for the keywords “calretinin and neuronal density and major depressive disorder,” one of which was a relevant study. We had two hits in PubMed for the keywords “calretinin and neuronal number and major depressive disorder,” one of which was a relevant study. We had 22 hits for the keywords “parvalbumin and neuronal density and schizophrenia,” of which six were relevant studies. We had 48 hits for the keywords “parvalbumin and neuronal number and schizophrenia, three of which were relevant studies.” We had two hits for the keywords “parvalbumin and neuronal density and bipolar disorder,” of which one was a relevant study. We had four hits for the keywords “parvalbumin and neuronal number and bipolar disorder,” two of which were relevant studies. We also searched the reference lists of published studies.

## Results

### Numbers of calretinin-immunoreactive neuron and neuronal densities in the septal nuclei of patients with schizophrenia, patients with affective disorders, and healthy control subjects

**Figure 2:**
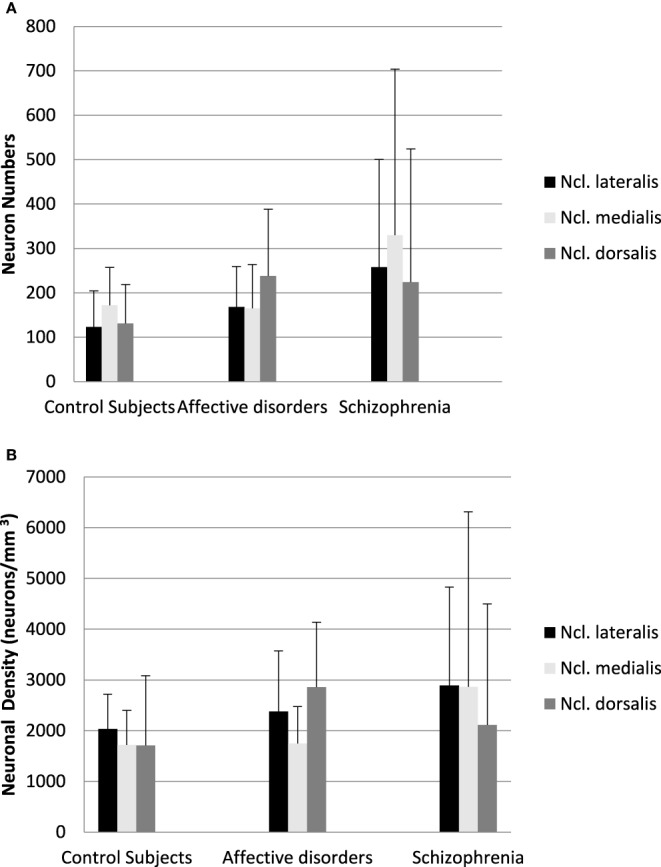
**(A)** Numbers of calretinin-immunoreactive neuron in the septal nuclei of patients with schizophrenia, patients with affective disorders, and healthy control subjects. The data are presented as the mean and standard deviation. **(B)** Densities of calretinin-immunoreactive neuron in the septal nuclei of patients with schizophrenia, patients with affective disorders, and healthy control subjects. The data are presented as the mean and standard deviation.

**Figure 3:**
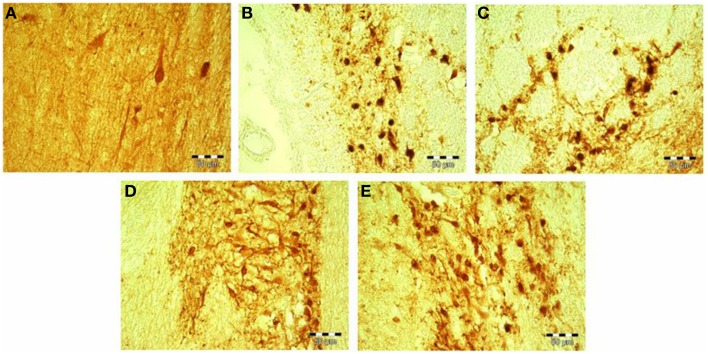
**(A–E)** Calretinin-immunopostive neurons in the Ncl. medialis of a healthy control subject **(A)**; Ncl. medialis of a patient with major depressive disorder **(B)**; Ncl. lateralis of a patient with major depressive disorder **(C)**; Ncl. medialis of a patient with schizophrenia **(D)**; and Ncl. medialis of a healthy control subject **(E)**. Scale bars correspond to 50 μm. Ncl., nucleus.

No significant differences were observed in the number of calretinin-immunoreactive neurons in the lateral (AFF vs. CTR, *P* = 0.48; SZ vs. CTR, *P* = 0.18; SZ vs. AFF, *P* = 0.78), medial (pars fimbrialis and pars intermedia; AFF vs. CTR, *P* = 1.00; SZ vs. CTR, *P* = 0.76; SZ vs. AFF, *P* = 0.88), and dorsal septal nuclei (AFF vs. CTR, *P* = 0.24; SZ vs. CTR, *P* = 0.69; SZ vs. AFF, *P* = 0.78) among patients with schizophrenia, patients with affective disorders and healthy control subjects (see Figures 2A, 3A–E; Table 2). There were no significant differences in the densities of calretinin-immunoreactive neurons in the lateral (AFF vs. CTR, *P* = 0.59; SZ vs. CTR, *P* = 0.61; SZ vs. AFF, *P* = 0.78), medial (pars fimbrialis and pars intermedia; AFF vs. CTR, *P* = 1.00; SZ vs. CTR, *P* = 0.88; SZ vs. AFF, *P* = 1.00), and dorsal septal nuclei (AFF vs. CTR, *P* = 0.24; SZ vs. CTR, *P* = 0.86; SZ vs. AFF, *P* = 0.22) among patients with schizophrenia, patients with affective disorders, and healthy control subjects (see Figure 2B; Table 2). The mean cross-sectional areas of the septal nuclei did not differ siginificantly among the patients with schizophrenia, patients with affective disorders, and healthy control subjects. The statistical power (1-β probability of error) was estimated for the *F*-tests used in the statistical analysis of number of cells and cell density, respectively. For the the dorsal septal nuclei, it was estimated as 0.612 and 0.736, for the medial septal nuclei (pars fimbrialis and pars intermedia) as 0.933 and 0.763, and for the lateral septal nuclei as 0.952 and 0.771. Therefore, it was concluded that our sample size would be large enough to detect statistically significant differences.

## Discussion

The present study demonstrates for the first time that there are no alterations in the density and number of calretinin-immunoreactive neurons in the lateral, medial, and dorsal septal nuclei of patients with schizophrenia or patients with affective disorders compared to healthy control subjects.

Although some studies demonstrated significant changes in calretinin-immunoreactive neurons in schizophrenia and bipolar disorder compared to control subjects in brain regions such as the dentate and the dorsolateral prefrontal gyri (Oh et al., 2012; Walton et al., 2012), the majority of studies of calretinin-immunoreactive neurons in schizophrenia in brain areas such as the hippocampus, prefrontal, and cingulate cortices, and the mammillary bodies (Zhang and Reynolds, 2002; Zhang et al., 2002; Bernstein et al., 2007; review by Eyles et al., 2002) and in affective disorders in the cingulate cortex (Cotter et al., 2002; see Table 3) are consistent with our present findings.

**Table 3:** **Post-mortem studies of calretinin-, calbindin-, and parvalbumin-immunoreactive neurons in patients with schizophrenia, bipolar patients, and patients with major depressive disorder compared to control subjects**.

**Authors**	**Sample sizes**	**Brain areas**	**Results**
Daviss and Lewis, 1995	*N* = 10 schizophrenia patients	Prefrontal cortical areas 9 and 46	Calbindin ↑ (schizophrenia patients compared to control subjects)
	*N* = 5 control subjects	Neuronal cell density	Calretinin ↔
Beasley and Reynolds, 1997	*N* = 18 schizophrenia patients	Prefrontal cortex	Parvalbumin ↓ (in layers III, IV of schizophrenia patients compared to control subjects)
	*N* = 22 control subjects	Neuronal cell density	
Kalus et al., 1997	*N* = 5 schizophrenia patients	Anterior cingulate cortex	Nissl ↔ (total neuronal density)
	*N* = 5 control subjects	Neuronal cell density	Parvalbumin ↑ (soma profile density in layers Va and Vb in schizophrenia patients compared to control subjects)
		Somal profile density	
Woo et al., 1997	*N* = 15 schizophrenia patients	Prefrontal cortex (areas 9 and 46)	Parvalbumin ↔
	*N* = 15 control subjects	Occipital cortex (area 17)	
		Neuronal cell density	
		Somal size	
Holt et al., 1999	*N* = 10 schizophrenia patients	Total striatum	Choline acetyltransferase↓ (in the total striatum and most prominent in the ventral striatum in schizophrenia patients compared to control subjects)
	*N* = 9 control subjects	Neuronal cell density	Calretinin ↓ (in the total striatum and most prominent in the caudate nucleus in schizophrenia patients compared to control subjects)
Reynolds and Beasley, 2001	*N* = 18 schizophrenia patients	Prefrontal cortex	Parvalbumin ↓ (in layers III, IV of schizophrenia patients compared to control subjects)
	*N* = 22 control subjects	Relative density of neurons	Calretinin ↔
Beasley et al., 2002	*N* = 15 schizophrenia patients	Neuronal cell density	Parvalbumin ↓ (in layer III of schizophrenia patients to control subjects)
	*N* = 15 bipolar patients		Calbindin ↓ (in layers II, III and V of schizophrenia patients compared to control subjects and layer V of bipolar patients compared to control subjects; by comparing individual laminar densities between groups (correction for multiple comparisons) only a reduction in layer II in schizophrenia patients compared to control subjects)
	*N* = 15 patients with major depressive disorder dorsolateral prefrontal cortex (Broadmann area 9)		Calretinin ↔
Cotter et al., 2002	*N* = 15 schizophrenia patients	Anterior cingulate cortex	Calretinin ↔
	*N* = 15 bipolar patients	Neuronal cell density	Parvalbumin ↔
	*N* = 15 patients with major depressive disorder	Neuronal cell size	Calbindin ↔
	*N* = 15 control subjects		
Reynolds et al., 2002	*N* = 15 schizophrenia patients	Entorhinal cortex	Parvalbumin ↓ (schizophrenia patients compared to control subjects)
		Prefrontal cortex	
	*N* = 15 bipolar patients	Neuronal cell density	Calbindin ↔
	*N* = 15 patients with major depressive disorder		Calretinin ↔
	*N* = 15 control subjects		
Zhang and Reynolds, 2002	*N* = 15 schizophrenia patients	Hippocampus	
	*N* = 15 bipolar patients	Relative cell density of neurons	Calretinin ↔
	*N* = 15 patients with major depressive disorder	Neuronal body size	Parvalbumin ↓ (cell density in male schizophrenia patients and bipolar patients (CA 1) compared to control subjects; neuronal body size ↓ in schizophrenia patients and bipolar patients compared to control subjects)
Zhang et al., 2002	*N* = 15 schizophrenia patients	Hippocampus	Parvalbumin ↓ (neuronal cell density)
	*N* = 15 control subjects	Neuronal cell density	
		Neuronal cell size	Calretinin ↔
Danos et al., 2003	*N* = 12 schizophrenia patients	Anteroventral thalamic nucleus (AN)	Parvalbumin ↓ (parvalbumin-immunoreactive-thalamocortical projection neurons in the left and right AN in schizophrenia patients compared to control subjects)
	*N* = 14 control subjects	Neuronal cell density	
Tooney and Chahl, 2004	*N* = 6 schizophrenia patients	Prefrontal cortex	Calretinin ↔
	*N* = 6 control subjects	Relative density of neurons	Calbindin ↔
		Somal size of neurons	Parvalbumin ↔
Chance et al., 2005	*N* = 12 schizophrenia patients	Planum temporale	Calbindin ↔
	*N* = 12 control subjects	Neuronal cell density	
Wheeler et al., 2006	*N* = 9 schizophrenia patients	Posterior cingulate cortex	Calbindin ↔
	*N* = 9 control subjects	Visual cortex	
		Neuronal cell density	
Bernstein et al., 2007	*N* = 15 schizophrenia patients	Mammillary bodies	Parvalbumin projection neurons ↓ (schizophrenia patients compared to control subjects)
	*N* = 15 control subjects	Neuronal cell number	Calretinin ↔
			GAD ↔
Pantazopoulos et al., 2007	*N* = 10 schizophrenia patients (SZ) *N* = 10 bipolar patients (BP) *N* = 16 control subjects (CS)	Entorhinal cortex (EC) Neuronal cell number Neuronal density Soma size	Parvalbumin ↓ (neuronal density and cell number in bipolar patients (BP) compared to control subjects (CS)) (neuronal density ↓ of the superficial layers of the lateral and caudal EC in BP compared to CS) (neuronal density ↓ of the superficial layers of the caudal EC in SZ compared to CS)
Rajkowska et al., 2007	*N* = 14 patients with major depressive disorder (MDD) *N* = 11 control subjects	Dorsolateral prefrontal cortex (dlPFC) Orbitofrontal cortex (ORB) Neuronal cell density Neuronal cell size	Calbindin ↓ in cell density and size in dlPFC in patients with MDD compared to control subjects a trend for reduction in parvalbumin and calbindin-immunoreactive neurons in cell density and cell size in ORB in patients with MDD compared to control subjects
Konradi et al., 2011a	*N* = 13 schizophrenia patients	Hippocampus	Parvalbumin ↓
	*N* = 20 control subjects	Neuron number	Somatostatin ↓
Konradi et al., 2011b	*N* = 14 bipolar patients	Hippocampus	Parvalbumin ↓ (CA4, CA1) and somatostatin ↓ (CA1) (bipolar patients compared to control subjects)
	*N* = 18 control subjects	Neuron number	
Wang et al., 2011	*N* = 11 patients with schizophrenia *N* = 13 patients with type 1 bipolar disorder *N* = 15 control subjects	Caudal entorhinal cortex (EC) Subiculum Parasubiculum Neuronal density	Parvalbumin ↓ and somatostatin ↓ (in the caudal EC and parasubiculum of bipolar and schizophrenia patients compared to control subjects Calbindin ↔
Oh et al., 2012	*N* = 15 patients with schizophrenia *N* = 15 bipolar patients *N* = 15 patients with major depressive disorder *N* = 15 control subjects	Dorsolateral prefrontal cortex (dlPFC) Neuronal cell density Neuronal cell size	Calretinin ↓ (in layer I in patients with major depressive disorder compared to control subjects) A significant correlation between reduced density of calretinin-immunoreactive in the dlPFC of patients with major depressive disorder and lower density or size of glial cells and pyramidal neurons in subjects from the Stanley Neuropathology Consortium
Walton et al., 2012	*N* = 15 control subjects *N* = 15 schizophrenia patients *N* = 15 bipolar patients *N* = 15 patients with major depressive disorder	Hippocampus Neuronal cell number	Calretinin ↑ (in the dentate gyrus of schizophrenia patients and bipolar patients compared to control subjects)

A novel interaction between calretinin and AMPA [(S)-2 amino-3-(3-hydroxy-5-methyl-4-isoazolyl)-propionic-acid] has been proposed as a potential target for the development of new antipsychotic therapeutics for schizophrenia (Siekmeier and vanMaanen, 2013). However, new drugs that affect this pathway should be developed with caution because the GABA-potentiating drug vigabatrin, an irreversible inhibitor of GABA-transaminase, induces alterations in GAD67, GAD65, parvalbumin, and calbindin levels in certain brain regions, including the hippocampus and cerebral cortex (Levav-Rabkin et al., 2010). Increased density of GAD65/67-immunoreactive neuropil suggests a GABAergic hyperactivity in the hippocampus and might compose a risk factor for suicidal behavior in affective disorders (Gos et al., 2009). Increased densities of GAD65/67-positive neurons in the dorsolateral prefrontal and superior temporal cortices and in the hippocampus have been observed among patients with major depressive disorder compared with control subjects and patients with bipolar disorder as well as in the orbitofrontal cortex among patients with major depressive disorder and bipolar disorder compared to control subjects (Bielau et al., 2007) and in the posterior subiculum and parahippocampal gyrus in treated patients with chronic schizophrenia compared with control subjects (Schreiber et al., 2011) Further, a reduced level of GAD67 protein in the prefrontal cortex has been observed in major depressive disorder compared with control subjects (Karolewicz et al., 2010), and a reduction in GAD67 along with parvalbumin-positive cells of the dorsal hippocampus has been observed in a rat model of schizophrenia (Dickerson et al., 2014). Calbindin/GAD67-positive and calretinin/GAD67-positive neurons are much more involved in pathological processes in brain diseases, including schizophrenia, than are neurons with GAD65 (Rocco et al., 2015). Alterations in GABAergic interneurons and minicolumns in the neocortex are characteristic of neuropathological diseases such as schizophrenia (Raghanti et al., 2010). Moreover, the dorsolateral prefrontal cortex, with its glutamatergic and GABAergic populations, has been the focus of recent schizophrenia research (Lewis et al., 2005; Hoftman and Lewis, 2011). Increased levels of GABA have been observed in prefrontal cortices in unmedicated patients with schizophrenia (Kegeles et al., 2012). Reduced numbers of parvalbumin-immunoreactive neurons in the hippocampus, prefrontal and frontal cortices, and mammillary bodies have been reported in patients with schizophrenia compared with control subjects (Beasley and Reynolds, 1997; Lewis et al., 2001; Reynolds and Beasley, 2001; Reynolds et al., 2001, 2002, 2004; Beasley et al., 2002; Zhang and Reynolds, 2002; Zhang et al., 2002; Bernstein et al., 2007; see Table 3) and in an animal model of schizophrenia (Reynolds et al., 2004; Penschuck et al., 2006; Harte et al., 2007; Bissonette et al., 2014). In addition, a significant decrease in parvalbumin-immunoreactive neurons in the entorhinal cortex of patients with bipolar disorder compared with control subjects has been observed (Pantazopoulos et al., 2007).

Increased oxidative stress and changes in antioxidant systems, such as decreased glutathione, in schizophrenia compromise the integrity of parvalbumin interneurons in the ventral hippocampus (Steullet et al., 2010).

Upregulation of parvalbumin in the prefrontal cortex during adolescence due to pre- and postnatal disturbances has been observed (Caballero et al., 2014). Age-related changes have been observed in the calbindin-, calretinin-, and parvalbumin-immunoreactive neurons of the human cerebral cortex (Bu et al., 2003) and in the parvalbumin-immunoreactive neurons of the medial and lateral geniculate nuclei of rhesus macaques (Gray et al., 2013). Nicotine enhances GABAergic and serotonin synaptic transmission in the medial septum (Wu et al., 2003; Aznar et al., 2005; DuBois et al., 2013).

Methylphenidate, which is used to treat children suffering from attention deficit hyperactivity disorder (ADHD), causes an increase in calretinin neurons in the medial septum and in the vertical limb of the diagonal band of Broca (MS/VDB) of rats (García-Avilés et al., 2015). Furthermore, cannabis abuse results in decreased expression of GAD67 in the parvalbumin-containing interneurons of the prefrontal cortex in a rat model of schizophrenia (Zamberletti et al., 2014). Prenatal lead exposure results in a loss of parvalbumin-interneurons co-labeled with GAD67 protein in specific brain regions such as medial prefrontal cortex, striatum, and hippocampus but to increased activity of the subcortical dopaminergic system and intensified locomotor response to cocaine in a rat model of schizophrenia (Stansfield et al., 2015).

A major limitation of the present study is the small numbers of healthy control subjects, patients with schizophrenia, and patients with affective disorders. It was not possible to determine the numbers of smokers and non-smokers in our study's population, a minor drawback of the study. The psychotropic medication in patients with schizophrenia and patients with affective disorders was only recorded during the last 3 months before the patients' lives.

Our findings of no significant differences in the density and number of immunoreactive calretinin neurons in the medial, lateral, and dorsal septal nuclei in patients with patients with schizophrenia, patients with affective disoders, and healthy control subjects should be interpreted as a preliminary result. Future research examining the distribution of calretinin neurons in the septal nuclei of patients with schizophrenia or patients with affective disorders should utilize larger sample sizes. Forthcoming investigations should also focus on the distribution and the densities of parvalbumin-immunoreactive neurons in the septal nuclei of patients with schizophrenia and patients with affective disorders compared with healthy control cases.

### Evolutionary trade-off of calretinin and schizophrenia

An evolutionary perspective on why calretinin is affected in the neurodevelopmental disorder schizophrenia provides some insight into this disorder. While several evolutionary theories have been proposed for the persistence of schizophrenia (Davis et al., 1991; Adriaens, 2008; da Silva Alves et al., 2008). Although it is impossible to verify these theories using ancestral hominin remains because brain tissue cannot be fossilized. Numerous hypotheses have been proposed for the superior cognitive abilities of *Homo species*, and these have been largely based on the threefold increase of the human neocortex during the Pleistocene period (2 Ma–13 kya). However, much of this research has focussed on brain anatomy in relation to cerebral volume size rather than neuro-hormonal regulation. It has been established that GABAergic interneurons are crucial in the diverse activities of pyramidal cells (Hendry et al., 1987; Zaitsev et al., 2009). From an evolutionary perspective, GABA has been shown to have an inhibitory function in the nervous systems of both vertebrates and invertebrates (Fiorillo and Williams, 1998; Gou et al., 2012). Although, it has been established that there exists a relationship between glutamatergic pyramidal neurons and GABAergic interneurons (of which calretinin-positive neurons are a subtype of GABAergic interneurons; Radonjic et al., 2014), this relationship is not well-understood in relation to the development of schizophrenia in humans.

### Evolution of the neurotransmitters gamma-aminobutyric acid and glutamate and their receptors

A recent theory hypothesizes that there may have been positive Darwinian selection in the modification of interneuron populations in humans, leading to cognitive specializations in *Homo species* (Sherwood et al., 2010). Current studies indicate that there is an evolutionary continuity between human and non-human primates, as well as changes in subcortical descending projections during human evolution (Rilling et al., 2008; Sherwood et al., 2010). Calretinin-positive interneurons are the most abundant GABAergic neurons in primates (Hladnik et al., 2014). Interestingly, anatomical and neurohormonal changes to the brain during human evolution may have informed changes in glutamatergic and GABAergic processes in interneurons, thereby possibly altering calretinin regulation in the hippocampus and in key motor centers. The development of obligate bipedalism required a host of morphological and neuro-hormonal changes: CNS, metabolic and cardiovascular responses due to sustained running, swivel hips, slow-twitch muscles, plantar arches and longer femurs, as well as modification of eccrine glands (Mattson, 2012). A recent theory contends that the advent of endurance hunting from *Homo erectus* onwards mediated thermo-regulatory changes in the dopaminergic system (Previc, 2002) and that such changes influenced neuro-hormonal regulation (Mattson, 2012). This theory also states that endurance hunting demanded retention of geographical areas and mnemonic recall in hippocampal areas to maximize resource acquisition (Mattson, 2012; Brisch et al., 2014). It could be suggested that the increase in physical activity levels (PAL) from *Homo erectus* onwards, may have caused an evolutionary trade-off in which higher metabolic demands in ancestral hominins may have come at an evolutionary cost of making GABA interneurons more vulnerable. Even slight physiologic variations in brain temperature may alter neuron composition and function (Andersen and Moser, 1995). Research indicates that GABA in the preoptic area and anterior hypothalamus (POAH) acts in heat regulation (Ishiwataa et al., 2005), while hyperthermia may increase hippocampal excitability and decrease GABA regulation in pyramidal cells (Qu et al., 2007; Qu and Leung, 2009). Therefore, selective pressures informing human brain evolution may have come at a cost of altering calretinin regulation of GABAergic hippocampal-interneurons, which may have contributed to the development of schizophrenia in humans.

## Funding

This study was supported by grants from the Stanley Medical Research Institute.

### Conflict of interest statement

The authors declare that the research was conducted in the absence of any commercial or financial relationships that could be construed as a potential conflict of interest.

## References

[B1] Adriaens P. R. (2008). Debunking evolutionary psychiatry's schizophrenia paradox. Med. Hypotheses 70, 1215–1222. 10.1016/j.mehy.2007.10.014 18226861

[B2] Andersen P. Moser E. I. (1995). Brain temperature and hippocampal function. Hippocampus 5, 491–498. 10.1002/hipo.450050602 8646277

[B3] Aznar S. Kostova V. Sorensen H. R. Knudsen G. M. (2005). A7 nicotinic receptor subunit is present on serotonin neurons projecting to hippocampus and septum. Synapse 55, 196–200. 10.1002/syn.20108 15635599

[B4] Baizer J. S. (2014). Unique features of the human brainstem and cerebellum. Front. Hum. Neurosci. 8:202. 10.3389/fnhum.2014.00202 24778611 PMC3985031

[B5] Barinka F. Druga R. (2010). Calretinin expression in the mammalian neocortex: a review. Physiol. Res. 59, 665–677. 20406030 10.33549/physiolres.931930

[B6] Baumann B. Danos P. Krell D. Diekmann S. Leschinger A. Stauch R.. (1999). Reduced volume of limbic system-affiliated basal ganglia in mood disorders: preliminary data from a postmortem study. J. Neuropsychiatry Clin. Neurosci. 11, 71–78. 10.1176/jnp.11.1.71 9990559

[B7] Beasley C. L. Reynolds G. P. (1997). Parvalbumin-immunoreactive neurons are reduced in the prefrontal cortex of schizophrenics. Schizophr. Res. 24, 349–355. 10.1016/S0920-9964(96)00122-3 9134596

[B8] Beasley C. L. Zhang Z. J. Patten J. Reynolds G. P. (2002). Selective deficit in prefrontal cortical GABAergic neurons in schizophrenia defined by the presence of calcium-binding proteins. Biol. Psychiatry 52, 708–715. 10.1016/S0006-3223(02)01360-4 12372661

[B9] Bernstein H. G. Krell D. Braunewell K. H. Baumann B. Gundelfinger E. D. Diekmann S.. (2001). Increased number of nitric oxide synthase immunoreactive Purkinje cells and dendate nucleus neurons in schizophrenia. J. Neurocytol. 30, 661–670. 10.1023/A:1016520932139 12118154

[B10] Bernstein H. G. Krause S. Krell D. Dobrowolny H. Wolter M. Stauch R.. (2007). Strongly reduced number of parvalbumin-immunoreactive projection neurons in the mammillary bodies in schizophrenia: further evidence for limbic neuropathology. Ann. N.Y. Acad. Sci. 1096, 120–127. 10.1196/annals.1397.077 17405923

[B11] Bielau H. Steiner J. Mawrin C. Trübner K. Brisch R. Meyer-Lotz G.. (2007). Dysregulation of GABAergic neurotransmission in mood disorders. Ann. N.Y. Acad. Sci. 1096, 157–169. 10.1196/annals.1397.081 17405927

[B12] Bissonette G. B. Bae M. H. Suresh T. Jaffe D. E. Powell E. M. (2014). Prefrontal cognitive deficits in mice with altered cerebral cortical GABAergic interneurons. Behav. Brain. Res. 259, 143–151. 10.1016/j.bbr.2013.10.051 24211452 PMC3874795

[B13] Blum B. P. Mann J. J. (2002). The GABAergic system in schizophrenia. Int. J. Neuropsychopharmacol. 5, 159–179. 10.1017/S1461145702002894 12135541

[B14] Brisch R. Bernstein H. G. Krell D. Dobrowolny H. Bielau H. Steiner J.. (2009). Dopamine-glutamate abnormalities in the frontal cortex associated with the catechol-O-methyltransferase (COMT) in schizophrenia. Brain Res. 1269, 166–175. 10.1016/j.brainres.2009.02.039 19268435

[B15] Brisch R. Bernstein H. G. Dobrowolny H. Krell D. Stauch R. Trübner K.. (2011). A morphometric analysis of the septal nuclei in schizophrenia and affective disorders: reduced neuronal density in the lateral septal nucleus in schizophrenia. Eur. Arch. Psychiatry Clin. Neurosci. 261, 47–58. 10.1007/s00406-010-0119-9 20607547

[B16] Brisch R. Saniotis A. Wolf R. Bielau H. Bernstein H. G. Steiner J.. (2014). The role of dopamine in schizophrenia from a neurobiological and evolutionary perspective: old fashioned, but still in vogue. Front. Psychiatry 5:47. 10.3389/fpsyt.2014.00047 24904434 PMC4032934

[B17] Bu J. Sathyendra V. Nagykery N. Geula C. (2003). Age-related changes in calbindin-D_28*k*_, calretinin, and parvalbumin-immunoreactive neurons in the human cerebral cortex. Exp. Neurol. 182, 220–231. 10.1016/S0014-4886(03)00094-3 12821392

[B18] Caballero A. Flores-Barrera E. Cass D. K. Tseng K. Y. (2014). Differential regulation of parvalbumin and calretinin interneurons in the prefrontal cortex during adolescence. Brain Struct. Funct. 219, 395–406. 10.1007/s00429-013-0508-8 23400698 PMC3665762

[B19] Cauli B. Zhou X. Tricoire L. Toussay X. Staiger J. F. (2014). Revisiting enigmatic cortical calretinin-expressing interneurons. Front. Neuroanat. 8:52. 10.3389/fnana.2014.00052 25009470 PMC4067953

[B20] Chance S. A. Walker M. Crow T. J. (2005). Reduced density of calbindin-immunoreactive interneurons in the planum temporale in schizophrenia. Brain Res. 1046, 32–37. 10.1016/j.brainres.2005.03.045 15927548

[B21] Cotter D. Landau S. Beasley C. Stevenson R. Chana G. MacMilian L.. (2002). The density and spatial distribution of GABAergic neurons, labelled using calcium binding proteins, in the anterior cingulate cortex in major depressive disorder, and schizophrenia. Biol Psychiarty 51, 377–386. 10.1016/S0006-3223(01)01243-4 11904132

[B22] Danos P. Baumann B. Krämer A. Bernstein H. G. Stauch R. Krell D.. (2003). Volumes of association thalamic nuclei in schizophrenia: a postmortem study. Schizophr. Res. 60, 141–155. 10.1016/S0920-9964(02)00307-9 12591578

[B23] da Silva Alves F. Figee M. van Avamelsvoort T. Veltman D. de Haan L. (2008). The revised dopamine hypothesis of schizophrenia: evidence from pharmacologcal MRI studies with atypical antipsychotic medication. Psychopharmacol. Bull. 41, 121–132. 10.1016/S0920-9964(08)70291-3 18362875

[B24] Daviss S. R. Lewis D. A. (1995). Local circuit neurons of the prefrontal cortex in schizophrenia: selective increase in the density of calbindin-immunoreactive neurons. Psychiatry Res. 59, 81–96. 10.1016/0165-1781(95)02720-3 8771223

[B25] Davis K. L. Kahn R. S. Ko G. Davidson M. (1991). Dopamine in schizophrenia: a review and reconceptualization. Am. J. Psychiatry 148, 1474–1486. 10.1176/ajp.148.11.1474 1681750

[B26] Deidda G. Bozarth I. F. Cancedda L. (2014). Modulation of GABAergic transmission in development and neurodevelopmental disorders: investigating physiology and pathology to gain therapeutic perspectives. Front. Cell. Neurosci. 8:119. 10.3389/fncel.2014.00119 24904277 PMC4033255

[B27] Dickerson D. D. Overeem K. A. Wolff A. R. Williams J. M. Abraham W. C. Bilkey D. K. (2014). Association of aberrant neural synchrony and altered GAD67 expression to maternal immune activation, a risk factor for schizophrenia. Transl. Psychiatry 4:e418. 10.1038/tp.2014.64 25072323 PMC4119228

[B28] DuBois D. W. Damborsky J. C. Fincher A. S. Frye G. D. Winzer-Serhan U. H. (2013). Varenicline and nicotine enhance GABAergic synaptic transmission in rat CA1 hippocampal and medial septum/diagonal band neurons. Life Sci. 92, 337–344. 10.1016/j.lfs.2012.12.013 23352971 PMC3598584

[B29] Eyles D. W. McGrath J. J. Reynolds G. P. (2002). Neuronal calcium-binding proteins and schizophrenia. Schizophr. Res. 57, 27–34. 10.1016/S0920-9964(01)00299-7 12165373

[B30] Fiorillo C. D. Williams J. T. (1998). Glutamate mediates an inhibitory postsynaptic potential in dopamine neurons. Nature 394, 78–82. 10.1038/27919 9665131

[B31] García-Avilés Á. Albert-Gascó H. Arnal-Vicente I. Elhaj E. Sanjuan-Arias J. Sanchez-Perez A. M. (2015). Acute oral administration of low doses of methylphenidate targets calretinin neurons in the rat septal area. Front. Neuroanat. 9:33. 10.3389/fnana.2015.00033 25852493 PMC4369875

[B32] González-Gómez M. Meyer G. (2014). Dynamic expression of calretinin in embryonic and early fetal human cortex. Front. Neuroanat. 8:41. 10.3389/fnana.2014.00041 24917793 PMC4042362

[B33] Gos T. Günther K. Bielau H. Dobrowolny H. Mawrin C. Trübner K.. (2009). Suicide and depression in the quantitative analysis of glutamic acid decarboxylase-immunoreactive neuropil. J. Affect. Disord. 113, 45–55. 10.1016/j.jad.2008.04.021 18538859

[B34] Gou Z. Wang X. Wang W. (2012). Evolution of neurotransmitter gamma-aminobutyric acid, glutamate and their receptors. Zool. Res. 33, E75–E81. 10.3724/SP.J.1141.2012.E05-06E75 23266985

[B35] Gray D. T. Rudolph M. L. Engle J. R. Recanzone G. H. (2013). Parvalbumin increases in the medial and lateral geniculate nuclei of aged rhesus macaques. Front. Aging Neurosci. 5:69. 10.3389/fnagi.2013.00069 24265617 PMC3821177

[B36] Harte M. K. Powell S. B. Swerdlow N. R. Geyer M. A. Reynolds G. P. (2007). Deficits in parvalbumin and calbindin immunoreactive cells in the hippocampus of isolation reared rats. J. Neural Trans. 114, 893–898. 10.1007/s00702-007-0627-6 17594127

[B37] Heldt S. A. Ressler K. J. (2006). Lessions of the habenula produce stress- and dopamine dependent alterations in prepulse inhibition in prepulse inhibition and locomotion. Brain Res. 1073–1074, 229–239. 10.1016/j.brainres.2005.12.053 PMC2561201 16442084

[B38] Hendry S. H. Schwark H. D. Jones E. G. Yan J. (1987). Numbers and proportions of GABA-immunoreactive neurons in different areas of monkey cerebral cortex. J. Neurosci. 7, 1503–1519. 3033170 10.1523/JNEUROSCI.07-05-01503.1987 PMC6568832

[B39] Hikosaka O. (2013). The habenula: from stress evasion to value-based decision-making. Nat. Rev. 11, 503–513. 10.1038/nrn2866 20559337 PMC3447364

[B40] Hladnik A. Džaja D. Damopil S. Jovanov-Miloševic N. Petanjek Z. (2014). Spatio-temporal extension in site of origin for cortical calretinin neurons in primates. Front. Neuroanat. 8:50. 10.3389/fnana.2014.00050 25018702 PMC4072090

[B41] Hoftman G. D. Lewis D. A. (2011). Postnatal development trajectories of neural circuits in the primate prefrontal cortex: Identifying sensitive periods for vulnerability to schizophrenia. Schizophr. Bull. 37, 493–503. 10.1093/schbul/sbr029 21505116 PMC3080694

[B42] Holt D. J. Herman M. M. Hyde M. Kleinman C. M. Sinton D. C. German L. B.. (1999). Evidence for a deficit in cholinergic interneurons in the striatum in schizophrenia. Neuroscience 94, 21–31. 10.1016/S0306-4522(99)00279-1 10613493

[B43] Horváth S. Palkovits M. (1987). Morphology of the human septal nuclei: a topographic atlas. Acta Morphol. Hung. 35, 157–174. 3137780

[B44] Joshi D. Fung S. J. Rothwell A. Weickert C. S. (2012). Higher gamma-aminobutyric acid neuron density in the white matter of orbital frontal cortex in schizophrenia. Biol. Psychiatry 72, 725–733. 10.1016/j.biopsych.2012.06.021 22841514

[B45] Inan M. Petros T. J. Anderson S. A. (2013). Losing your inhibition: linking cortical GABAergic interneurons to schizophrenia. Neurobiol. Dis. 53, 36–48. 10.1016/j.nbd.2012.11.013 23201207 PMC3593807

[B46] Inta D. Lima-Ojeda J. M. Lau T. Tang W. Dorman C. Sprengel R.. (2013). Electroconvulsive therapy induces neurogenesis in frontal rat brain areas. PLoS ONE 8:e69869. 10.1371/journal.pone.0069869 23922833 PMC3724733

[B47] Ishiwataa T. Saitoa T. Hasegawac H. Yazawaa T. Kotanid Y. Otokawae M.. (2005). Changes of body temperature and thermoregulatory responses of freely moving rats during GABAergic pharmacological stimulation to the preoptic area and anterior hypothalamus in several ambient temperatures. Brain Res. 1048, 32–40. 10.1016/j.brainres.2005.04.027 15913569

[B48] Kalus P. Senitz D. Beckmann H. (1997). Altered distribution of parvalbumin-immunoreactive local circuit neurons in the anterior cingulate cortex of schizophrenic patients. Psychiatry Res. 75, 49–59. 9287373 10.1016/s0925-4927(97)00020-6

[B49] Karolewicz B. Maciag D. O'Dwyer G. Stockmeier C. A. Feyissa A. M. Rajkowska G. (2010). Reduced level of Glutamic Acid Decarboxylase-67 kDa in the prefrontal cortex in major depression. Int. J. Neuropsychopharmacol. 13, 411–420. 10.1017/S1461145709990587 20236554 PMC2857696

[B50] Kegeles L. S. Mao X. Stanford A. D. Girgis R. Ojeil N. Xu X.. (2012). Elevated prefrontal cortex γ-aminobutyric acid and glutamate-glutamine levels in schizophrenia measured *in vivo* with proton magnetic resonance spectroscopy. Arch. Gen. Psychiatry 69, 449–460. 10.1001/archgenpsychiatry.2011.1519 22213769

[B51] Konradi C. Yang C. K. Zimmerman E. I. Lohmann K. M. Gresch P. Pantazopoulos H.. (2011a). Hippocampal interneurons are abnormal in schizophrenia. Schizophr. Res. 131, 165–173. 10.1016/j.schres.2011.06.007 21745723 PMC3159834

[B52] Konradi C. Zimmerman E. I. Yang C. K. Lohmann K. M. Gresch P. Pantazopoulos H.. (2011b). Hippocampal interneurons are abnormal in bipolar disorder. Arch. Gen. Psychiatry 68, 340–350. 10.1001/archgenpsychiatry.2010.175 21135314 PMC3197787

[B53] Kostovic I. Judãs M. Sedmak G. (2011). Developmental history of the subplate zone, subplate neurons and interstitial white matter neurons: relevance for schizophrenia. Int. J. Dev. Neurosci. 29, 193–205. 10.1016/j.ijdevneu.2010.09.005 20883772

[B54] Koyama Y. Hattori T. Shimzu S. Taniguchi M. Yameda K. Takamura H.. (2013). DBZ (DISC1-binding zinc finger protein)-deficient mice display abnormalities in basket cells in the somatosensory cortices. J. Chem. Neuroanat. 53, 1–10. 10.1016/j.jchemneu.2013.07.002 23912123

[B55] Lecourtier L. de Vasconcelos A. P. Cosquer B. Cassel J. C. (2010). Combined lesions of GABAergic and cholinergic septal neurons increase locomotor activity and potentiate the locomotor response to amphetamine. Behav. Brain Res. 213, 175–182. 10.1016/j.bbr.2010.04.050 20450937

[B56] Levav-Rabkin T. Melamed O. Clarke G. Faber M. Cryan J. F. Dinan T. G.. (2010). A sensitive period of mice inhibitory system to neonatal GABA enhancement by Vigbatrin is brain region dependent. Neuropsychopharmacology 35, 1138–1154. 10.1038/npp.2009.219 20043003 PMC3055404

[B57] Lewis D. A. Hashimoto T. Volk D. W. (2005). Cortical inhibitory neurons and schizophrenia. Nat. Rev. Neurosci. 6, 312–324. 10.1038/nrn1648 15803162

[B58] Lewis D. A. Cruz D. A. Melchitzky D. S. Pierri J. N. (2001). Lamina specific deficits in parvalbumin-immunoreactive varicosities in the prefrontal cortex of subjects with schizophrenia. Am. J. Psychiatry 158, 1411–1422. 10.1176/appi.ajp.158.9.1411 11532725

[B59] Ma J. Tai S. K. Leung L. S. (2012). Septohippocampal GABAergic neurons mediate the altered behaviors induced by N-methyl-D-aspartate receptor antagonists. Hippocampus 22, 2208–2218. 10.1002/hipo.22039 22592894

[B60] Mattson M. P. (2012). Evolutionary aspects of human exercise–born to run purpose-fully. Ageing Res. Rev. 11, 347–352. 10.1016/j.arr.2012.01.007 22394472 PMC3356485

[B61] Mikkonen M. Soininen H. Pitkanen A. (1997). Distribution of parvalbumin-, calretinin-, and calbindin-D28-immunoreactive neurons and fibres in the human entorhinal cortex. J. Comp. Neurol. 388, 64–88. 9364239

[B62] Nitsch R. Ohm T. G. (1995). Calretinin immunoreactive structures in the human hippocampal formation. J. Comp. Neurol. 360, 475–487. 10.1002/cne.903600309 8543653

[B63] Oh D. H. Son H. Hwang S. Kim S. H. (2012). Neuropathological abnormalities of astrocytes, GABAergic neurons, and pyramidal neurons in the dorsolateral prefrontal cortices of patients with major depressive disorder. Eur. Neuropharmacol. 22, 330–338. 10.1016/j.euroneuro.2011.09.001 21962915

[B64] Pantazopoulos H. Lange N. Baldessarini R. J. Berrreta S. (2007). Parvalbumin neurons in the entorhinal cortex of subjects diagnosed with bipolar disorder or schizophrenia. Biol. Psychiatry 61, 640–652. 10.1016/j.biopsych.2006.04.026 16950219 PMC1964505

[B65] Pennington K. Dicker P. Cotter D. R. (2008). Evidence for reduced neuronal somal size within the insular cortex, but not in affective disorders. Schizophr. Res. 106, 164–177. 10.1016/j.schres.2008.08.022 18805671

[B66] Penschuck S. Flagstad P. Didriksen M. Leist M. Michael-Titus A. T. (2006). Decrease in parvalbumin-expressing neurons in the hippocampus and increased phencyclidine-induced locomotor activity in the rat methylazoxymethanol (MAM) model of schizophrenia. Eur. J. Neurosci. 23, 279–284. 10.1111/j.1460-9568.2005.04536.x 16420437

[B67] Previc F. H. (2002). Thyroid hormone production in chimpanzees and humans: implications for the origins of human intelligence. Am. J. Phys. Anthropol. 118, 402–403. 10.1002/ajpa.10095 12124921

[B68] Qu L. Liu X. Wu C. Leung L. S. (2007). Hyperthermia decreases GABAergic synaptic transmission in hippocampal neurons of immature rats. Neurobiol. Dis. 27, 320–327. 10.1016/j.nbd.2007.06.003 17643307

[B69] Qu L. Leung L. S. (2009). Effects of temperature elevation on neuronal inhibition in hippocampal neurons of immature and mature rats. J. Neurosci. Res. 87, 2773–2785. 10.1002/jnr.22105 19396879

[B70] Radonjic N. V. Ortega J. A. Memi F. Dionne K. Jakovcevski I. Zecevic N. (2014). The complexity of the calretinin-expressing progenitors in the human cerebral cortex. Front. Neuroanat. 8:82. 10.3389/fnana.2014.00082 25165435 PMC4131197

[B71] Raghanti M. A. Spocter M. A. Butti C. Hof P. R. Sherwood C. C. (2010). A comparative perspective on minicolumns and inhibitory GABAergic interneurons in the neocortex. Front Neuroanat. 4:3. 10.3389/neuro.05.003.2010 20161991 PMC2820381

[B72] Rajkowska G. O'Dwyer G. Teleki Z. Stockmeier C. A. Miguel-Hidalgo J. J. (2007). GABAergic neurons immunoreactive calcium binding proteins are reduced in the prefrontal cortex in major depression. Neuropsychopharmacology 32, 471–482. 10.1038/sj.npp.1301234 17063153 PMC2771699

[B73] Ranft K. Dobrowolny H. Krell D. Bielau H. Bogerts B. Bernstein H. G. (2010). Evidence for structural abnormalities of the human habenular complex in affective disorders but not in schizophrenia. Psychol. Med. 4, 557–567. 10.1017/S0033291709990821 19671211

[B74] Reynolds G. P. Beasley C. L. (2001). GABAergic neuronal subtypes in the human frontal cortex-developments and deficits in schizophrenia. J. Chem. Neuroanat. 1–2, 95–100. 10.1016/S0891-0618(01)00113-2 11470557

[B75] Reynolds G. P. Zhang Z. J. Beasley C. L. (2001). Neurochemical correlates of cortical GABAergic deficits in schizophrenia: selective losses of calcium binding protein immunoreactivity. Brain Res. Bull. 55, 579–584. 10.1016/S0361-9230(01)00526-3 11576754

[B76] Reynolds G. P. Beasley C. L. Zhang Z. J. (2002). Understanding the neuropathology of schizophrenia: selective deficits of subtypes of cortical GABAergic neurons. J. Neural. Transm. 109, 881–889. 10.1007/s007020200072 12111475

[B77] Reynolds G. P. Abdul-Manim Z. Neill J. C. Zhang Z. J. (2004). Calcium binding protein markers of GABA deficits in schizophrenia-postmortem studies and animal models. Neurotoxicol. Res. 6, 57–61. 10.1007/BF03033297 15184106

[B78] Rilling J. K. Glasser M. F. Preuss T. M. Ma X. Zhao T. Hu X.. (2008). The evolution of the arcuate fasciculus revealed with comparative DTI. Nat. Neurosci. 11, 426–428. 10.1038/nn2072 18344993

[B79] Rocco B. R. Sweet R. A. Lewis D. A. Fish K. N. (2015). GABA-synthesis enzymes in calbindin and calretinin neurons in monkey prefrontal cortex. Cereb. Cortex. 10.1093/cercor/bhv051. [Epub ahead of print]. PMC4830294 25824535

[B80] Sandyk R. (1992). Pineal and habenula calcification in schizophrenia. Int. J. Neurosci. 67, 19–30. 10.3109/00207459208994773 1305634

[B81] Savitz J. B. Nugent A. C. Bogers W. Roiser J. B. Bain E. E. Neumeister A.. (2011a). Habenula volume in bipolar disorder and major depressive disorder: a high resolution MRI study. Biol. Psychiatry 69, 336–343. 10.1016/j.biopsych.2010.09.027 21094939 PMC3030670

[B82] Savitz J. B. Bonne O. Nugent A. C. Vythillingham M. Bogers W. Charney D. S.. (2011b). Habenula volume in post-traumatic stress disorder measured with high-resolution MRI. Biol. Mood Anxiety Disord. 1:7. 10.1186/2045-5380-1-7 22738208 PMC3384261

[B83] Schreiber S. Bernstein H. G. Friedirch R. Stauch R. Ketzler B. Dobrowolny H. (2011). Increased density of GAD 65/67 immunoreactive neurons in the posterior and parahippocampal gyrus in treated patients with schizophrenia. World J. Psychiatry 12, 57–61. 10.3109/15622975.2010.539270 21250934

[B84] Sherwood C. C. Raghanti M. A. Stimpson C. D. Spocter M. A. Uddin M. Boddy A. M.. (2010). Inhibitory interneurons of the human prefrontal cortex display conservedevolution of the phenotype and related genes. Proc. R. Soc. B 277, 1011–1020. 10.1098/rspb.2009.1831 19955152 PMC2842764

[B85] Shin R. Kobayashi K. Hagihara H. Koyan J. H. Miyake S. Tajinda K.. (2013). The immature dentate gyrus represents a shared phenotype of mouse models of epilepsy and psychiatric disease. Bipolar Disord. 15, 405–421. 10.1111/bdi.12064 23560889 PMC3752967

[B86] Siekmeier P. J. vanMaanen D. P. (2013). Development of antipsychotic medications with novel mechanisms of action based on computational modeling of hippocampal neuropathology. PLoS ONE 8:e58607. 10.1371/journal.pone.0058607 23526999 PMC3602393

[B87] Spampanato J. Sullivan R. K. Turpin F. R. Bartlett P. F. Sah P. (2012). Properties of doublecortin expressing neurons in the adult mouse dentate gyrus. PLoS ONE 7:e41029. 10.1371/journal.pone.0041029 22957010 PMC3434174

[B88] Stansfield K. H. Ruby K. N. Soares B. D. McGlothan J. L. Liu X. Guilarte T. R. (2015). Early life lead exposure recapitulates the selective loss of parvalbumin-positive GABAergic interneurons and subcortical dopamine system hyperactivity present in schizophrenia. Trans Psychiatry 5:e522. 10.1038/tp.2014.147 25756805 PMC4354343

[B89] Steullet P. Cabungcal J. H. Kulak A. Kraftsik R. Chen Y, Dalton, T. P.. (2010). Redox dysregulation affects the ventral but not dorsal hippocampus: impairement of parvalbumin neurons, gamma oscillations, and related behaviors. J. Neurosci. 30, 2547–2558. 10.1523/JNEUROSCI.3857-09.2010 20164340 PMC6634545

[B90] Suárez-Solá M. L. González-Delgado F. J. Pueyo-Morlans M. Medina-Bolivar O. C. Hernández-Acosta N. C. González-Gómez M.. (2009). Neurons in the white matter of the adult human neocortex. Front Neuroanat. 3:7. 10.3389/neuro.05.007.2009 19543540 PMC2697018

[B91] Sutherland R. J. (1982). The dorsal diencephalic conduction system: a review of the anatomy and functions of the habenular complex. Neurosci. Biobehav. Rev. 6, 1–13. 10.1016/0149-7634(82)90003-3 7041014

[B92] Tanti A. Westphal W. P. Girault V. Brizard B. Devers S. Leguisquet A.-M.. (2013). Region-dependent and stage-specific effects of stress, environmental enrichment, and antidepressant treatment on hippocampal neurogenesis. Hippocampus 23, 797–811. 10.1002/hipo.22134 23592526

[B93] Todkar K. Scotti A. L. Schwaller B. (2012). Absence of the calcium-binding protein calretinin, not of calbindin D28k, causes a permanent impairment of murine adult hippocampal neurogenesis. Front. Mol. Neurosci. 5:56. 10.3389/fnmol.2012.00056 22536174 PMC3332231

[B94] Tooney P. A. Chahl L. A. (2004). Neurons expressing calcium-binding proteins in the prefrontal cortex in schizophrenia. Prog. Neuropsychopharmacol. Biol. Psychiatry 28, 273–278. 10.1016/j.pnpbp.2003.10.004 14751422

[B95] Walløe S. Pakkenberg B. Fabricius K. (2014). Stereological estimation of total cell numbers in the human cerebral and cerebellar cortex. Front. Hum. Neurosci. 8:508. 10.3389/fnhum.2014.00508 25076882 PMC4097828

[B96] Walton N. M. Zhou Y. Kogan J. H. Shin R. Webster M. Gross A. K.. (2012). Detection of an immature dendate gyrus feature in schizophrenia/bipolar patients. Transl. Psychiatry 2, e135. 10.1038/tp.2012.56 22781168 PMC3410619

[B97] Wang A. Y. Lohmann K. M. Yang K. Zimermann E. I. Pantazopoulos H. Herring N.. (2011). Bipolar disorder type 1 are accompanied by decreased density of parvalbumin- and somatostatin-positive interneurons in the parahippocampal region. Acta. Neuropathol. 122, 615–626. 10.1007/s00401-011-0881-4 21968533 PMC4207060

[B98] Wheeler D. G. Dixon G. Harper C. G. (2006). No difference in calcium-binding protein immunoreactivity in the posterior cingulate and visual cortex: Schizophrenia and controls. Prog. Neuro. Psycho. Biol. Psychiatry 30, 630–639. 10.1016/j.pnpbp.2005.11.091 16503370

[B99] Woo T. U. Miller J. L. Lewis D. A. (1997). Schizophrenia and the parvalbumin-containing class of cortical circuit neurons. Am. J. Psychiatry 154, 1013–1015. 9210755 10.1176/ajp.154.7.1013

[B100] Wu M. Hajszan T. Leranth C. Alreja M. (2003). Nicotine recruits a local glutamatergic circuit to excite septohippocampal GABAergic neurons. Eur. J. Neurosci. 18, 1155–1168. 10.1046/j.1460-9568.2003.02847.x 12956714

[B101] Yang Y. Fung S. J. Rothwell A. Tianmei S. Weickert C. S. (2011). Increased interstitial white matter neuron density in the dorsolateral prefrontal cortex of people with schizophrenia. Biol. Psychiatry 69, 63–70. 10.1016/j.biopsych.2010.08.020 20974464 PMC3005941

[B102] Zaitsev A. V. Povysheva N. V. Gonzalez-Burgos G. Rotaru D. Fish K. N. Krimer L. S.. (2009). Interneuron diversity in layers 2–3 of monkey prefrontal cortex. Cereb. Cortex 19, 1597–1615. 10.1093/cercor/bhn198 19015370 PMC2693619

[B103] Zamberletti E. Beggiato S. Steardo L. Antonelli T. Ferraro L. Rubino T.. (2014). Alterations of prefrontal cortex GABAergic transmission in the complex psychotic-like phenotypes induced by adolescent delta-9-terahydrocannibol exposure in rats. Neurobiol. Dis. 63, 35–47. 10.1016/j.nbd.2013.10.028 24200867

[B104] Zhang Z. J. Reynolds G. P (2002). A selective decrease in the relative density of parvalbumin-immunoreactive neurons in the hippocampus in schizophrenia. Schizophr. Res. 55, 1–10. 10.1016/S0920-9964(01)00188-8 11955958

[B105] Zhang Z. Sun J. Reynolds G. P. (2002). A selective reduction in the relative density of parvalbumin-immunoreactive neurons in the hippocampus in patients with schizophrenia. Chin. Med. J. 115, 819–823. 12123544

